# *Drosophila pachea* asymmetric lobes are part of a grasping device and stabilize one-sided mating

**DOI:** 10.1186/s12862-016-0747-4

**Published:** 2016-09-01

**Authors:** Flor T. Rhebergen, Virginie Courtier-Orgogozo, Julien Dumont, Menno Schilthuizen, Michael Lang

**Affiliations:** 1 Naturalis Biodiversity Center, Darwinweg 2, 2333 CR Leiden, The Netherlands; 2 Institute for Biodiversity and Ecosystem Dynamics, University of Amsterdam, Science Park 904, 1098 XH Amsterdam, The Netherlands; 3 Institute Biology Leiden, Leiden University, Sylviusweg 72, 2333 BE Leiden, The Netherlands; 4 Team “Évolution des drosophiles”, Institut Jacques Monod, CNRS, UMR 7592, Université Paris Diderot, Sorbonne Paris Cité, 15 rue Hélène Brion, 75013 Paris, France; 5 Team “Division cellulaire et reproduction”, Institut Jacques Monod, CNRS, UMR 7592, Université Paris Diderot, Sorbonne Paris Cité, 15 rue Hélène Brion, 75013 Paris, France

**Keywords:** *Drosophila pachea*, *Nannoptera* species group, Left-right asymmetry, Epandrial lobe, Mating position, Genitalia, Laser ablation, Micro-surgery

## Abstract

**Background:**

Multiple animal species exhibit morphological asymmetries in male genitalia. In insects, left-right genital asymmetries evolved many times independently and have been proposed to appear in response to changes in mating position. However, little is known about the relationship between mating position and the interaction of male and female genitalia during mating, and functional analyses of asymmetric morphologies in genitalia are virtually non-existent. We investigated the relationship between mating position, asymmetric genital morphology and genital coupling in the fruit fly *Drosophila pachea*, in which males possess an asymmetric pair of external genital lobes and mate in an unusual right-sided position on top of the female.

**Results:**

We examined *D. pachea* copulation by video recording and by scanning electron microscopy of genital complexes. We observed that the interlocking of male and female genital organs in *D. pachea* is remarkably different from genital coupling in the well-studied *D. melanogaster*. In *D. pachea*, the female oviscapt valves are asymmetrically twisted during copulation. The male’s asymmetric lobes tightly grasp the female’s abdomen in an asymmetric ‘locking’ position, with the left and right lobes contacting different female structures. The male anal plates, which grasp the female genitalia in *D. melanogaster*, do not contact the female in *D. pachea*. Experimental lobe amputation by micro-surgery and laser-ablation of lobe bristles led to aberrant coupling of genitalia and variable mating positions, in which the male was tilted towards the right side of the female.

**Conclusion:**

We describe, for the first time, how the mating position depends on coupling of male and female genitalia in a species with asymmetric genitalia and one-sided mating position. Our results show that *D. pachea* asymmetric epandrial lobes do not act as a compensatory mechanism for the change from symmetric to one-sided mating position that occurred during evolution of *D. pachea*’s ancestors, but as holding devices with distinct specialized functions on the left and right sides.

**Electronic supplementary material:**

The online version of this article (doi:10.1186/s12862-016-0747-4) contains supplementary material, which is available to authorized users.

## Background

A recurrent feature of the external morphology of genitalia of many animal species is left-right asymmetry, which has evolved many times independently from bilateral symmetry [1–3]. However, our evolutionary and functional understanding of morphological asymmetry in genitalia remains very limited [1, 2]. This is somewhat surprising, given the fact that the evolution of animal genitalia has recently been under close investigation [4–7] and the fact that the function and evolution of secondarily evolved morphological asymmetries in other body parts has been extensively studied [8–17].

Several hypotheses for the evolution of asymmetric genitalia have been put forward, including space constraints, ecological pressures, antagonistic male–female co-evolution and changes in mating positions [1, 2, 18, 19]. Few of these have enjoyed sufficient support to be generally embraced, except maybe Huber's hypothesis that genitalia may become asymmetric in response to evolutionary changes in mating position [18]. According to this hypothesis, the shift from an ancestral symmetric mating position to a fixed one-sided mating position occurred first during evolution and then led to the evolution of genital morphological asymmetry although it is probably more realistic to imagine that male behavior, female preference and genitalia co-evolve constantly. A more complex formulation of Huber’s hypothesis would be that the first asymmetry that evolves is behavioral, which consequentially leads to multiple successive evolutionary changes in morphology and behavior which strengthen the asymmetry. In this scenario, the evolutionary appearance of a fixed one-sided copulation position may affect genital function in at least three non-mutually exclusive ways: (1) right and left sides may change to morphologically compensate for the mismatch resulting from asymmetric contact between male and female genitalia; (2) right and left sides may start to assume different functions; (3) one side may lose its function and becomes reduced.

One-sided mating can evolve from symmetric mating via sexual selection or via neutral evolution. Evolution of one-sided mating behaviour by sexual selection could occur if males that mate one-sided gain greater fertilization success than males that mate symmetrically, which could happen through several non-mutually exclusive mechanisms. First, females may prefer to copulate with males that mate one-sided (precopulatory sexual selection by female choice [20]). Second, when mating one-sided, the male may bypass certain female structures that usually prevent sperm from entering the reproductive tract when mating symmetrically (postcopulatory sexual selection by sexually antagonistic conflict [21–23]). Third, one-sided mating may stimulate the female in a way that activates its receptivity and thus increases fertilization, egg quality or egg laying rate (postcopulatory sexual selection by cryptic female choice [5, 21, 24]).

Even though the hypothesis that genital asymmetry evolves in response to changes in mating position could be tested through detailed analyses of the function of asymmetric genitalia in species that recently evolved a one-sided mating position, we know of only one example relating evolutionary patterns of copulatory behaviour to the evolution of genital asymmetry [25–27]. Male earwigs (Dermaptera) in the suborder Forficulina usually possess two laterally paired penises, but use only one of them for transferring sperm during copulation. In the forficuline clade Eudermaptera, the left penis has become degenerated and only a single functional penis remains [25, 27]. Interestingly, in *Labidura riparia,* a forficuline earwig species that is closely related to the Eudermaptera, males preferentially use their right penis, even though they possess two fully functional penises [25]. These observations suggest that the degeneration of the left penis in the Eudermaptera was preceded by preferential use of the right penis for copulation. However, it remains unclear whether the apparent left-sided reduction in penis number in Eudermaptera is related to changes in mating position because males *Labidura riparia* turn clockwise or anticlockwise towards a tail-to-tail configuration during copulation, independently of which penis they use [25]. So far, the earwig case study does not represent unequivocal evidence that changes in mating position could drive the evolution of genital asymmetry. However, it does show that the evolution of morphological asymmetry in the genitalia can be preceded by the evolution of one-sided mating behaviour.

Alternatively, one-sided mating behaviour could be a consequence rather than the cause of the evolution of genital asymmetry. Currently no studies support this hypothesis directly, but an experimental study on *Drosophila melanogaster* (Diptera: Drosophilidae) has shown that creating an artificial asymmetry in genital morphology can cause an asymmetry in mating position [28]. Male *D. melanogaster* adopt a symmetric mating position on top of the female. Ablation of a mechanosensory bristle on the male’s left genital clasper led to mating positions in which the male was bent towards the right side of the female, and ablation of a mechanosensory bristle on the male’s right genital clasper produced mating positions in which the male was bent towards the left side of the female [28]. This study shows that the appearance of a new asymmetry in genital morphology can cause a novel asymmetry in mating position, at least in a laboratory setting. In conclusion, very little is known about the evolutionary relationships between asymmetry in mating position and asymmetry in genital morphology.

Changes in genital asymmetry and mating postures might possibly also evolve neutrally in some cases. Praying mantid males (Mantodea) possess large left-right asymmetric genital structures [29] and copulate while positioned on top of the female along the female midline, with the male abdomen twisted laterally around the female abdomen so that the male genitalia contacts the female genitalia from below in a so called ‘false-male-above’ mating position [30]. The male abdomen twists either clockwise or counter-clockwise and the direction is correlated with male genital asymmetry, which can occur as ‘dextral’ or ‘sinistral’ chirality morphs [31–34]. Both male morphs are observed in *C. baldersoni*, and the positioning of either genital structures onto the female genital organs has been described through scanning electron microscopy analyses [32]. Interestingly, no functional difference has been observed apart from formation of two chirally different genitalia complexes and both male morphs revealed similar levels of mating success [32].

In Diptera, at least eight different mating positions have been recorded and in all of them the male genitalia are inversely positioned relative to the female genitalia: the dorsal surface of the aedeagus (phallus) contacts the ventral side of the female reproductive tract [19, 35–41]. In seven species of the *Drosophila melanogaster* subgroup, mating positions are reported to be male-above and symmetric [28, 35, 36]. In these species, the male flexes its abdomen around the posterior end of the female abdomen during mating, allowing the whole posterior surface of the male external genitalia to come into close contact with the posterior surface of the female genitalia. In this configuration, the dorsal side of the male epandrium contacts the ventral side of the female genitalia and the dorsally positioned male anal plates grasp and hold the ventral sides of the female oviscapt valves, thereby accomplishing a typical inversed male–female genital complex [35, 38–40]. The interaction of male and female genitalia during copulation has, to our knowledge, never been described in dipteran species with asymmetric genitalia or one-sided mating positions, so it is currently unclear whether such species would not follow the inverse coupling of male and female genitalia as observed in the *melanogaster* subgroup.

In the one-sided male-above mating positions, male and female genitalia may contact each other in several ways. First, male and female genitalia may contact each other in a symmetric fashion with male and female genitalia positioned inversely relative to each other, as observed in species of the *melanogaster* subgroup. In this case, the one-sided mating position could be accomplished through lateral flexion of the male abdomen, causing an asymmetry in the overall position of the male body. Second, male and female genitalia may contact each other asymmetrically, and the one-sided mating position could be a consequence of asymmetric coupling of male and female genitalia and the male’s inability to bend its abdomen laterally. In this case, the microscale angle between male and female genitalia should reflect the macroscale angle between male and female bodies during copulation. Third, the one-sided mating position could be a consequence of both lateral flexion of the male abdomen and asymmetric contact between male and female genitalia. In this case, the microscale difference in the dorso-ventral axes of male and female genitalia would not necessarily be the same as the macroscale angle between male and female bodies. Distinguishing between these three possibilities is important, because the hypothesis that genitalia evolve morphological asymmetries in response to changes in mating position rests on the assumption that evolutionarily derived mating positions are associated with asymmetric coupling of male and female genitalia. Therefore, we decided to examine the precise position of the male and female genitalia during copulation of *D. pachea* [42], a species that has asymmetric external male genitalia and that copulates in a right-sided mating position [43].

The *nannoptera* species group in the genus *Drosophila* is an emerging and promising model system to investigate the mechanisms underlying the evolution of genital asymmetries and genital interactions during copulation [43, 44]. The *nannoptera* group comprises four described species, and three of them exhibit left-right asymmetries in male genitalia. *Drosophila acanthoptera* has an asymmetric aedeagus [45, 46], *D. wassermanni* possesses asymmetric anal plates, as well as an asymmetric aedeagus [45, 46] and *D. pachea* displays a conspicuous left-right asymmetry in the morphology of the epandrium: it has two lobes, the left lobe being about 1.5 times as long as the right lobe [43]. In contrast, *D. nannoptera* has symmetric genitalia like most other drosophilids [45] and is presumably the sister group of the other three *nannoptera* group species [44]. Furthermore, in one laboratory stock of *D. pachea*, 20 % of the males possess short symmetric epandrial lobes, probably due to a mutation that appeared during the rearing of the stock in the laboratory [43].

Previously, we found that *D. pachea* has an asymmetric mating position that is unusual among drosophilids [43]. During copulation, males are positioned on top of the female and are oriented towards the female’s right wing: at 4–11 min after the beginning of copulation, the male’s antero-posterior body axis is positioned at an angle of 8.55 ± 1.79 (mean ± SD) degrees towards the right side of the female’s antero-posterior body axis. Interestingly, mutant male *D. pachea* with short symmetric lobes still mate right-sided, but with an angle that is more variable than in the wild-type [43]. Some of the symmetric mutant males (39 %, *n =* 9/23) fell off shortly after mounting the female and are not able to form a stable mating complex at all [43]. These observations suggest that the asymmetric epandrial lobes may mechanically stabilize the male in a right-sided angle on top of the female, or may provide sensory input that enables the male to sense the female’s reproductive structures and find its way onto the female’s dorsum. However, the precise interaction of *D. pachea* male and female genitalia during copulation has not yet been investigated.

Importantly, the symmetric mutants do not only differ from the wild-type in the symmetry of the length of the epandrial lobes, but also in the distribution of bristles on the lobes. In wild-type males, lobe bristles are evenly distributed throughout the distal parts of both lobes, while they are combined into a single patch in symmetric mutant males [43]. Therefore, the possibility remains that the abnormal mating position of the symmetrical mutants is not (only) caused by the altered length of the epandrial lobes, but rather (or also) by the difference in bristle distribution, which could impair the sensing of female’s reproductive structures or the stabilization of the mating complex. Furthermore, the abnormal mating position of the symmetrical mutants could be a consequence of yet another, unknown phenotypic effect of the mutation underlying the epandrial lobe symmetry [43]. Hypothetically, this mutation could also affect internal genital structures, sensory organs or even brain structures in such a way that mating behaviour might be altered. Therefore, to accurately determine the function of the epandrial lobes, we decided to experimentally modify epandrial lobe length and epandrial bristle distributions in *D. pachea* wild-type males and examine whether such manipulations affect copulation.

Only a limited number of previous studies have used experimental modification of insect genitalia to study the function of genital traits, presumably because the microscopic size of insect genital structures hampers manual surgical manipulation (but see references [25, 28, 47–50]. In the past 5 years, however, ablation by precision laser surgery, a method developed by Polak and Rashed [51], has emerged as a reliable approach to modify insect genitalia, and it has been applied to study the function of genital spines in *Callosobruchus maculatus* [52], *Drosophila ananassae* [53, 54] and *D. bipectinata* [51, 55] and of sex combs in *D. melanogaster* and *D. bipectinata* [56]*.* Importantly, laser ablation makes it possible to precisely modify microscopic genital structures without damaging neighbouring tissues.

In this study, we explored the relationship between one-sided mating position, asymmetric genital morphology and genital coupling in *D. pachea* by video recording of mating behavior and scanning-electron-microscopy of male–female genital coupling. In particular, we wanted to test if surgical manipulation of *D. pachea* male asymmetric lobes would affect formation and maintenance of the coupling of male and female genitalia during mating. To investigate this, we amputated the lobes by manual micro-surgery, we ablated lobe bristles with a laser and we analyzed symmetric mutant males.

## Results

### Ablation or shaving of the left epandrial lobe does not affect the mating sequence and its duration

To assess the role of the left epandrial lobe, we either cut off part of the left lobe in wild-type males with surgical scissors under CO_2_-anaesthesia (hereafter referred to as ‘left lobe cut’ treatment) or we ablated all the bristles covering the left lobe with our laser ablation set-up (hereafter referred to as ‘left lobe shaved’ treatment). We examined mating behaviour 4–14 days after surgery by video recording of courtship and copulation of single virgin couples. Unmodified wild-type males were processed as ‘left lobe cut’ males except that lobes were not cut. We did not explicitly control for side-effects of surgical wounding, as ablating another part of the body was expected to alter mating behaviour as well and would thus not constitute a relevant control.

**Fig. 1:**
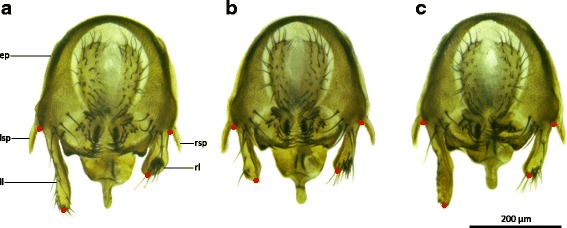
The epandrial lobe treatments are precise and organ-specific. Dissected epandria of a control wild-type male (**a**), a ‘left lobe cut’ male (**b**) and a ‘left lobe shaved’ male (**c**). The length of the epandrial lobes was calculated as the distance between the base and the tip of the epandrial lobes (*red dots*). Abbreviations: ep, epandrium; ll, left epandrial lobe; rl, right epandrial lobe; lsp, left epandrial spine; rsp, right epandrial spine. Scale bar is 200 μm

After video recording, male epandria were dissected to assess the extent of the surgery. No damage could be detected on neighbouring organs and in most cases a small part of the left lobe still remained (Fig. 1a-b). We estimated the length of the lobes of ‘left lobe cut’ males, ‘left lobe shaved’ males and unmodified males by measuring the distance between the distal tip and the proximal base of each lobe, where it merges with a lateral spine into the epandrium (Fig. 1a–c). In ‘left lobe cut’ males, the length of the remainder of the left lobe was 114.85 ± 33.88 μm (mean ± SD; *n =* 18). This was not significantly different from the length of the right lobe, which was estimated to be 130.49 ± 6.10 μm (mean ± SD; *n =* 18; Welch’s *T*-test: *T*_*18.10*_ = 1.93, *P =* 0.070; Table 1), but was significantly shorter than the length of the left lobe in unmodified males, which was estimated to be 185.53 ± 12.46 μm (mean ± SD; *n =* 16; Welch’s *T*-test: *T*_*22.26*_ = −8.21, *P <* 0.001; Table 1). The length of the left lobe was not different in ‘left lobe shaved’ males (*n =* 18) and unmodified males (*T*-test: *T*_*31*_ = 0.46, *P =* 0.647; Table 1). Mortality among the ‘left lobe cut’ males was not higher than mortality among unmodified males (in fact lower, but not significantly so: Fisher’s exact test, *P =* 0.347; Table 2). Mortality among ‘left lobe shaved’ males was also not higher than mortality among unmodified males (in fact lower, but not significantly so: Fisher’s exact test, *P =* 0.314; Table 2).

**Table 1:** Epandrial lobe lengths of *D. pachea* males used for movie analysis (mean ± SD)

Treatment	Left lobe length [μm]	Right lobe length [μm]	*n*
*Left lobe cut*	114.85 ± 33.88	130.49 ± 6.10	18
*Left lobe shaved*	187.81 ± 15.28	127.59 ± 13.42	18
*Unmodified (wild-type)*	185.53 ± 12.46	130.10 ± 9.32	16

**Table 2:** Mating behaviour and mating position in wild-type *D. pachea* couples with unmodified males, ‘left lobe cut’ males and ‘left lobe shaved’ males

	Unmodified wild-type	Left lobe cut	Left lobe shaved	*Wild-type in Lang & Orgogozo 2012* [24]	*Symmetric mutant in Lang & Orgogozo 2012* [24]
*Total number of couples*	16	18	18	*19*	*22*
*Number of couples that failed to copulate*	0	1	0	*0*	*8*
*Number of males that did copulate, but failed to do so at first attempt*	0	2	2	*0*	*3*
*Time until male courtship started [min:sec; mean ± SD]*	9:53 ± 13:12 (*n =* 16)	5:58 ± 9:41 (*n =* 17)	9:06 ± 14:35 (*n =* 18)	*10:56 ± 26:39 (n = 19)*	*1:52 ± 1:45 (n = 22)*
*Duration of male courtship until copulation occurred [min:sec; mean ± SD]*	9:38 ± 8:15 (*n =* 16)	20:18 ± 25:07 (*n =* 16)	12:13 ± 32:03 (*n =* 18)	*6:15 ± 7:15 (n = 19)*	*13:30 ± 20:04 (n = 14)*
*Duration of copulation until female started kicking [min:sec; mean ± SD]*	17:11 ± 8:00 (*n =* 14)	14:21 ± 4:17 (*n =* 14)	13:28 ± 3:58 (*n =* 14)	*not recorded*	*not recorded*
*Total duration of copulation [min:sec; mean ± SD]*	27:38 ± 6:46 (*n =* 16)	27:35 ± 6:22 (*n =* 17)	25:28 ± 5:20 (*n =* 18)	*34:55 ± 7:08 (n = 19)*	*39:55 ± 10:56 (n = 14)*
*Mating angle at 10–15 min after copulation starts (present study) or at 4–11 min* [24] *[degree; mean ± SD]*	6.06 ± 3.64 (*n =* 12)	−1.98 ± 11.13 (*n =* 12)	8.87 ± 11.70 (*n =* 14)	*8.55 ± 1.79 (n = 12)*	*5.77 ± 5.33 (n = 11)*
*Tilting index at 10–15 min after copulation starts [mean ± SD]*	0.960 ± 0.038 (*n =* 12)	0.880 ± 0.070 (*n =* 12)	0.909 ± 0.096 (*n =* 14)	*not recorded*	*not recorded*
*Male mortality between date of treatment and date of video recording or snap-freezing (including males used for snap-freezing experiments)*	26.9 % (*n =* 14/52)	18.0 % (*n =* 9/50)	17.1 % (*n =* 6/35)		

Data from a previous study [43] are incorporated. Note that courtship time is higher in left lobe cut males than in control males but that this difference is not significant (log-transformed; LM: *F*_*2,47*_ = 1.70, *P =* 0.194). Courtship duration is the time interval between the moment of first apparent male courtship behaviour and the moment at which the male mounts the female. SD = standard deviation, *n =* number of observations

Couples of *D. pachea* showed a stereotyped sequence of behaviours prior to copulation, which resembled the standard *Drosophila* sequence of courtship behaviour [57]. Males first showed courtship wing-flicking behaviour, and then started to tap the female’s genitalia with their forelegs while still wing-flicking and licking the genitalia of the female with the proboscis; the female finally everted her oviscapt and allowed the male to mount her and copulate. In unmodified control males, pre-copulatory wing-flicking behaviour started 9:53 ± 13:12 min:seconds (mean ± SD, *n =* 16, Additional file 1) after the males were introduced to the female in the mating cell; copulation followed 9:38 ± 8:15 min (mean ± SD, *n =* 16) after the start of wing-flicking behaviour. The mean timing of the start of wing-flicking behaviour was not significantly affected by treatment (log-transformed; LM: *F*_*2,48*_ = 0.60, *P >* 0.5; Table 2), nor was the mean duration of wing-flicking behaviour until copulation occurred (log-transformed; LM: *F*_2,47_ = 1.70, *P =* 0.194; Table 2). All males that copulated showed pre-copulatory wing-flicking behaviour, except one ‘left lobe cut’ male.

All 16 unmodified males managed to form a long-lasting (>5 min) mating complex at the first copulation attempt. Most ‘left lobe cut’ males (15/18) and ‘left lobe shaved’ males (16/18) also formed a durable copulation complex successfully at the first attempt. Of the ‘left lobe cut’ males that failed at the first attempt (3/18), two were successful at the second attempt and one male attempted to copulate 5 times and still was not successful after 1 h. Both ‘left lobe shaved’ males that were not able to mount the female at the first attempt succeeded at the second attempt. Copulation lasted 27:38 ± 6:46 min (mean ± SD, *n =* 16) in couples with untreated males, and the duration of copulation was not significantly affected by treatment (Kruskal-Wallis test: *K*_*2*_ = 2.82, *P =* 0.244; Table 2).

In couples with unmodified males, the females started to move and kick the male repeatedly with their hind legs after 17:11 ± 8:00 min (mean ± SD, *n =* 14) of copulation. The females then continued kicking the male in irregular bouts of kicking activity until copulation ended; the end of copulation was typically the result of a bout of female kicking behaviour. The starting time of female kicking behaviour was unaffected by treatment (LM: *F*_*2,39*_ = 1.62, *P =* 0.217; Table 2), as was the remaining duration of copulation after the female had started kicking (LM: *F*_*2,39*_ = 0.13, *P =* 0.878). Not all females showed kicking behaviour: 4 females coupled to a ‘left lobe shaved’ male, 3 females coupled to a ‘left lobe cut’ male and 2 females coupled to an unmodified male did not kick the male, and in these cases copulation ended while the female was standing still.

### Ablation of the left epandrial lobe makes the male tilt to the right side of the female during copulation and increases variance in mating angle

**Fig. 2:**
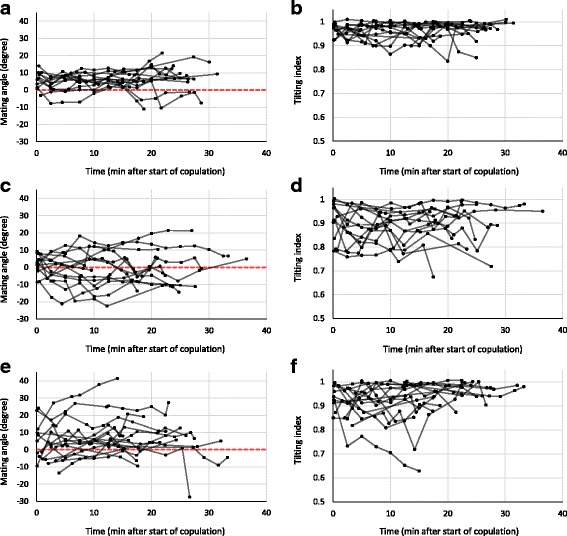
Mating angle and tilting index are affected by left lobe ablation and by laser removal of left lobe bristles. Shown are time series of mating angle (**a**, **c**, **e**) and tilting index (**b**, **d**, **f**) in mating couples with control unmodified control males (**a**, **b**), “left lobe cut” males (**c**, **d**) and “left lobe shaved” males (**e**, **f**). Measurements from the same couple are connected by a *black* line. No measurements were taken if the female could not be observed from exactly above, so the length of a time series does not necessarily represent mating duration

Our video recordings of unmodified wild-type *D. pachea* (*n =* 16) showed a stereotypical mating position as previously described [43]: after mounting the female, the male typically positioned his antero-posterior body axis in a clockwise angle relative to the antero-posterior body axis of the female. This angle varied somewhat among copulating couples and over time as copulation proceeded (Fig. 2a). The variation among couples appeared to be lowest at 10–15 min into copulation, at which the mating angle was estimated to be 6.06 ± 3.64° (mean ± SD, *n =* 12; Table 2, Fig. 2a, Additional files 1 and 2). This mating angle did not differ significantly from the mean mating angle that we estimated from the raw data of our previous study [43] in wild-type males after 10–15 min of copulation (unpublished data; *T*-test: *T*_*20*_ = 1.84, *P =* 0.080). The variance in mating angles was not equal between treatments (Bartlett’s test: *K*^*2*^_*2*_ = 13.15, *P =* 0.001). The variance in mating angles after 10–15 min of copulation was significantly larger in mating couples with a ‘left lobe cut’ male than in mating couples with unmodified males (Bonferroni-corrected F-test: *F*_*11*_*,*_*11*_ = 9.40, *P =* 0.003; Fig. 2c).

**Fig. 3:**
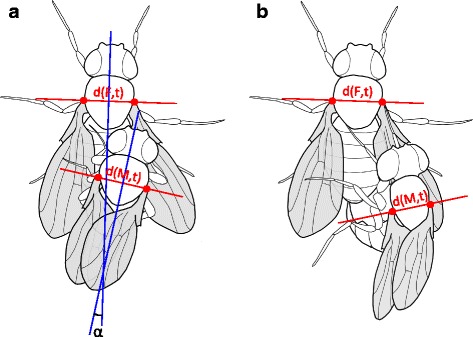
Measures of mating angle and tilting index in two different mating positions. **a** Tilting index and mating angle measurement protocols of this study (*red lines*) and Lang & Orgogozo 2012 (*blue lines*). The resulting angle α is the same in the two methods, as the *blue* lines are perpendicular to the *red* lines. Tilting index (see Materials and Methods section) approximates 1 when the male is positioned exactly on top of the female, as the measured distance between the bases of the wings of the male (denoted by *d(M,t)*) is similar to the measured distance between the bases of the wings of the female (denoted by *d(F,t)*), corrected for the difference in size between male and female. **b** Tilting results in a negative mating angle and in a decreased (<1) tilting index, as the measured distance between the bases of wings of the male *d(M,t)* is shorter than the measured distance between the bases of wings of the female *d(F,t)*

During copulation, unmodified males typically positioned themselves right on top of the female whereas ‘left lobe cut’ males tended to slide down laterally on the right side of the female. As the position of a male’s antero-posterior body axis was estimated based on the position of a male’s thorax and wings (Fig. 3a-b), the mean mating angle after 10–15 min of copulation in couples with ‘left lobe cut’ males was estimated to be negative (towards the left side of the female,−1.98 ± 11.13°, mean ± SD, *n =* 12; Table 2; Fig. 2c), although males were sliding down the female’s right side during copulation. This shows that the mating angle can only be an informative measure of right-side-oriented mating behaviour when the male is positioned on top of the female’s abdomen. To quantify the degree to which the male was tilted towards the female’s right side during copulation, we defined a new measure that we called ‘tilting index’. This measure, which ranges between 0 and 1, takes values close to 1 when the male is not tilted relative to the female, and decreasing values as the male slides further downwards on one side of the female. This ‘sliding down’ always occurred on the right side of the female, never on the left side.

The tilting index approximated 1 in mating couples with unmodified males, although it was slightly variable over time and between mating couples (Fig. 2b). After 10–15 min of copulation, tilting index of mating couples with unmodified males was estimated at 0.960 ± 0.038 (mean ± SD, *n =* 12; Table 2). Tilting index of mating couples with ‘left lobe cut’ males was estimated to be 0.880 ± 0.070 (mean ± SD, *n =* 12; Table 2). A linear model revealed that treatment significantly affected mean tilting index (arcsine-transformed; LM: *F*_*2,35*_ = 4.36, *P =* 0.022), and a post-hoc test showed that mean tilting index in mating couples with a ‘left lobe cut’ male was significantly lower than the mean tilting index in mating couples with an unmodified male (Tukey HSD: *P =* 0.016). This result indicates that ‘left lobe cut’ males tended to slide down more on the right side of the female than unmodified males did (Fig. 2d). Tilting index variance did not differ among treatments after arcsine-transformation (Bartlett’s test: *K*^*2*^_*2*_ = 4.23, *P =* 0.121).

**Fig. 4:**
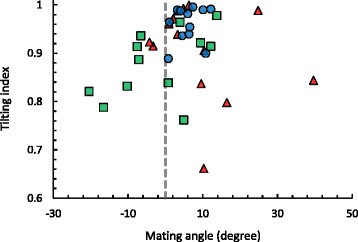
Average mating angles and tilting indices at 10–15 min into copulation. Extreme mating angles and tilting indices occur more often in ‘left lobe cut’ males (*green squares*) and ‘left lobe shaved’ males (*red triangles*) than in untreated control males (*blue circles*) at 10–15 min into copulation. Mating angle correlates with tilting index in ‘left lobe cut’ males, while this is not the case in ‘left lobe shaved’ males

Tilting index was significantly positively correlated with mating angle after 10–15 min of copulation in mating couples with ‘left lobe cut’ males (Spearman’s rank test: *ρ*_10_ = 0.59, *P =* 0.049; Fig. 4), showing that negative mating angles with ‘left lobe cut’ males did indeed occur when the male’s abdomen was sliding down the female’s right side (Fig. 3b). This correlation did not exist in mating couples with unmodified males (Spearman’s rank test: *ρ*_10_ = 0.36, *P =* 0.246; Fig. 4).

### Ablation of bristles on the left epandrial lobe increases variance in mating angle

The mating angle in mating couples with ‘left lobe shaved’ males was estimated to be 8.87 ± 11.70° (mean ± SD, *n =* 14; Table 2) after 10–15 min of copulation, and was not significantly different from the mating angle in couples with unmodified males (Welch’s *T*-test: *T*_*15.85*_ = 0.85, *P =* 0.407). However, the variance in mating angles was significantly larger in mating couples with ‘left lobe shaved’ males than in mating couples with unmodified males (Bonferroni-corrected F-test: *F*_*13,11*_ = 10.38, *P =* 0.001; Fig. 2a, e). There was no significant difference in variance in mating angles between mating couples with ‘left lobe shaved’ and ‘left lobe cut’ males (Bonferroni-corrected F-test: *F*_*11,13*_ = 0.91, *P >* 0.5).

Tilting index in mating couples with ‘left lobe shaved’ males was 0.909 ± 0.096 (mean ± SD, *n =* 14; Table 2) after 10–15 min of copulation, and did not differ significantly from mean tilting index in mating couples with unmodified males (Tukey HSD: *P =* 0.257) and mating couples with ‘left lobe cut’ males (Tukey HSD: *P =* 0.339). However, tilting indices in some of the mating couples with ‘left lobe shaved’ males were as low or even lower than tilting indices in mating couples with ‘left lobe cut’ males (Fig. 2f), indicating that a few ‘left lobe shaved’ males did not stay on top of the female during copulation but slid downwards. Tilting index was not correlated with mating angle after 10–15 min of copulation in mating couples with ‘left lobe shaved’ males (Spearman’s rank test: *ρ*_12_ = −0.30, *P =* 0.302; Fig. 4).

### Mating position does not change with epandrial lobe length

We tested whether variation in mating position within treatment groups could be explained by differences in lobe lengths or body size, by testing whether mating angle or tilting index after 10–15 min of copulation was correlated with a) left or right epandrial lobe length, b) the sum of both epandrial lobe lengths, c) the ratio of left and right epandrial lobe lengths and d) tibia length (Table 3). In mating couples with unmodified males, neither mating angle nor tilting index was correlated with left epandrial lobe length, the sum of left and right lobe lengths, the ratio of lobe lengths or tibia length (Table 3). In a dataset containing the present data and previously published data on wild-type *D. pachea* mating angles and lobe lengths [43], there was no significant correlation between left or right lobe length and mating angle (Table 3). In conclusion, there were no significant correlations between mating angle or tilting index and lobe lengths or tibia length in mating couples with ‘left lobe cut’ and ‘left lobe shaved’ males (Table 3). This shows that within-treatment variation in mating position could not be explained by differences in epandrial lobe length or body size.

**Table 3:** Correlation of genital morphology with mating angle and tilting index, at 10–15 min after the start of copulation

Treatment		Mating angle	Tilting index
*Unmodified wild-type (n = 11)*	Left epandrial lobe length	*ρ =* −0.02, *P >* 0.5	*ρ =* −0.15, *P >* 0.5
	Right epandrial lobe length	*ρ =* 0.78, *P =* 0.007	*ρ =* 0.32, *P =* 0.341
	Sum of left and right lobe lengths	*ρ =* 0.33, *P =* 0.327	*ρ =* 0.29, *P =* 0.386
	Ratio of left and right lobe lengths	*ρ =* −0.54, *P =* 0.089	*ρ =* −0.30, *P =* 0.369
	Tibia length	*ρ =* −0.03, *P >* 0.5	*ρ =* 0.51, *P =* 0.094
*Unmodified wild-types from present study combined with data from* [24] *(n = 23)*	Left epandrial lobe length	*ρ =* 0.06, *P >* 0.5	*not recorded by Lang & Orgogozo (2012)*
	Right epandrial lobe length	*ρ =* 0.36, *P =* 0.088	
	Sum of left and right lobe lengths	*ρ =* 0.17, *P =* 0.445	
	Ratio of left and right lobe lengths	*ρ =* −0.26, *P =* 0.222	
*Left lobe cut (n = 12)*	Left epandrial lobe length	*ρ =* 0.38, *P =* 0.218	*ρ =* 0.22, *P =* 0.500
	Right epandrial lobe length	*ρ =* −0.29, *P =* 0.366	*ρ =* −0.34, *P =* 0.276
	Sum of left and right lobe length	*ρ =* 0.28, *P =* 0.379	*ρ =* 0.13, *P >* 0.5
	Ratio of left and right lobe lengths	*ρ =* 0.47, *P =* 0.128	*ρ =* 0.34, *P =* 0.287
	Tibia length	*ρ =* 0.33, *P =* 0.297	*ρ =* 0.39, *P =* 0.210
*Left lobe shaved (n = 14)*	Left epandrial lobe length	*ρ =* −0.50, *P =* 0.069	*ρ =* 0.19, *P >* 0.5
	Right epandrial lobe length	*ρ =* −0.41, *P =* 0.146	*ρ =* 0.26, *P =* 0.365
	Sum of left and right lobe lengths	*ρ =* −0.49, *P =* 0.081	*ρ =* 0.31, *P =* 0.281
	Ratio of left and right lobe lengths	*ρ =* 0.18, *P >* 0.5	*ρ =* −0.20, *P =* 0.482
	Tibia length	*ρ =* −0.08, *P >* 0.5	*ρ =* 0.20, *P >* 0.5

Spearman’s correlation coefficients and associated *P*-values are presented for five explanatory variables

### In *D. pachea* mating complexes, only the ventral part of the male genitalia contacts the female

**Fig. 5:**
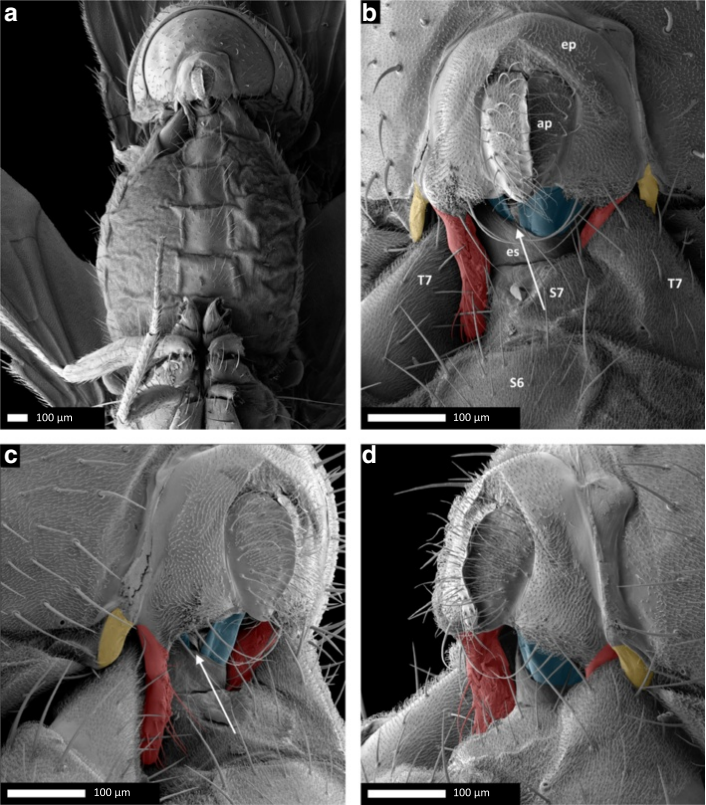
Scanning electron micrographs of coupled male and female *D. pachea* genitalia at 10 min after the beginning of copulation. **a** 37× and **b** 190× magnification of a ventral view, (**c**) left lateral view, (**d**) right lateral view. Male: ap, anal plates; ep, epandrium. Female: es, eversible sheath (intersegmental membrane between 7th sternite and oviscapt valves); S6, 6th sternite, S7, 7th sternite; T7, 7th tergite. The male’s epandrial lobes are artificially coloured in *red*, the male’s lateral epandrial spines in *yellow* and the female’s oviscapt valves in *blue*. The medial gap between the female’s oviscapt valves is indicated by an arrow and is visible from the left side, but not from the right side. Scale bar is 100 μm

To characterize the relative position of the male and female genital structures during copulation, we snap-froze *D. pachea* mating couples 10 min after the initiation of copulation, when the mating angle variation of unmodified males was lowest, and examined the interaction of male and female genitalia by scanning electron microscopy. We found that the dorso-ventral plane of the male epandrium was positioned perpendicularly to the dorso-ventral axis of the female genitalia and that only the ventral structures of the male genitalia contacted the female (Fig. 5a–d, Additional file 3). The dorsal part of the male genitalia, including the anal plates and the genital arch, did not touch the female genitalia. The extremity of the female abdomen was found to be grasped by the ventral male genital structures: the epandrial lobes, the lateral spines, the aedeagus, and possibly also the surstyli/claspers, which were not visible on SEM micrographs (Fig. 5b–d) because they were bent dorsally towards the eversible sheath of the female oviscapt valves and were hidden by the ventral male epandrium.

### Asymmetric epandrial lobes in wild-type *D. pachea* grasp the female abdomen in a stereotyped locking system

**Fig. 6:**
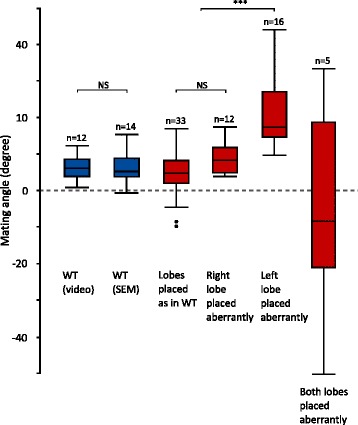
Comparison of the macroscale and microscale mating angles. Mating angles are shown for the wild-type (*blue*), with first the macroscale angle (based on video analysis) and second the microscale angle (based on SEM). Microscale angles are also presented for all treatments (left lobe shaved, right lobe shaved, left lobe cut, right lobe cut, symmetric mutant) combined together (*red*), but separated according to the positioning of the lobes. NS: not significant, ***: *P <* 0.001

In all 14 examined mating couples of unmodified wild-type *D. pachea*, male epandrial lobes and epandrial spines were positioned on the female in the same way (Fig. 5a–d, Additional file 3). The male’s long left epandrial lobe was pressed on the female’s left ventral side near the ventral edge of the female’s 7th tergite, which covers a large part of the female’s posterior ventral region lateral of the 7th sternite (terminology adapted from Grimaldi (1987) and Markow and O’Grady (2006) [58, 59]). The male’s left epandrial spine was located on the dorsal left side of the female’s 7th tergite. The left side of the female’s 7th tergite was clamped between the male’s left epandrial lobe and its left epandrial spine, thus ‘locking’ the female’s abdomen between epandrial lobe and epandrial spine. The male’s right lobe was pressed against the eversible sheath of the female’s oviscapt and was positioned on the female’s ventral right side, with its tip positioned underneath the posterior edge of the female’s 7th tergite. The male’s right epandrial spine was found on the dorsal side of the female’s 7th tergite, with the right side of the female’s 7th tergite clamped between the male’s right epandrial lobe and right epandrial spine, much like the left side of the female. The female abdomen was therefore tightly ‘locked’ between the male’s epandrial lobe and epandrial spine both on the left side and on the right side (Fig. 5). The angle between the female abdomen and the male epandrium was estimated to be 6.09 ± 4.38° (mean ± SD, *n =* 14; Fig. 6; Table 4). This angle did not differ significantly from the macroscale mating angle measured 10–15 min after the start of copulation based on our video recordings (Welch’s *T*-test: *T*_*23.98*_ = 0.02, *P =* 0.985; Fig. 6).

**Table 4:** Placement of epandrial lobes, mating position and visibility of oviscapt valves in SEM-analysis of *D. pachea* mating couples

Treatment	Total number of mating couples	Number of mating couples with both lobes in ‘wild-type position’	Number of mating couples with abnormal left lobe position	Number of mating couples with abnormal right lobe position	Number of mating couples with abnormal position of both lobes	Microscale angle of male relative to female (degrees; mean ± SD)	Number of mating couples in which oviscapt valves were visible
*Unmodified wild-type*	14	14	0	0	0	6.09 ± 4.40^+^	14
*Symmetric mutant*	16	6	6	3	1	9.77 ± 12.36	9
*Left lobe cut*	14	3	8	2	1	16.68 ± 10.45*^+^	7
*Right lobe cut*	10	5	0	4	1	1.15 ± 18.77	8
*Left lobe shaved*	4	3	1	0	0	10.68 ± 14.00	3
*Right lobe shaved*	8	3	0	3	2	1.39 ± 16.38*	6

The microscale angle of the male epandrium relative to the female abdomen was measured from the ventral side (Fig. 10); values denoted by * are significantly different from each other at α = 0.05, as are values denoted by +

### The female oviscapt is twisted asymmetrically during copulation

**Fig. 7:**
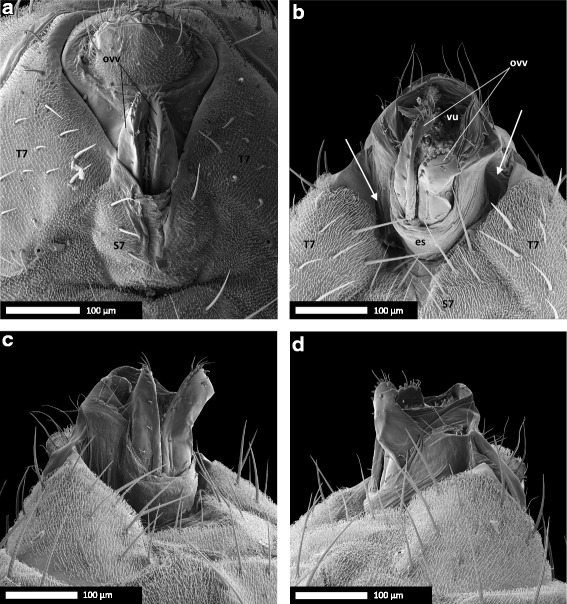
Female oviscapt valves are asymmetrically twisted during copulation and symmetric in resting position. **a** Genitalia of a *D. pachea* female in resting position. **b** Genitalia of a *D. pachea* female at 10 min after the start of copulation, with the male removed, in ventral view; **c** in left lateral view and **d** in right lateral view. Note the impressions left by the male’s epandrial lobes, lateral of the oviscapt, in panels **b**–**d** (arrows). Abbreviations: es, eversible sheath (intersegmental membrane between 7th sternite and oviscapt valves); ovv, oviscapt valves; S7, 7th sternite; T7, 7th tergite; vu, vulva. Scale bar is 100 μm

In resting position, the oviscapt was retracted, with the oviscapt valves extending from beneath the medially split 7th sternite and no asymmetry was apparent on SEM of female external genitalia (*n =* 8; Fig. 7a). At 10 min into copulation, the female’s 7th sternite, which is medially split, was spread apart and the female oviscapt was everted distally. The oviscapt valves were often partially visible in SEM of mating couples. We observed that the oviscapt valves, whenever visible, were invariably twisted asymmetrically in a clockwise fashion. This rendered the medial gap between the oviscapt valves visible from the left side of the female, but not from the right side (Fig. 5b–d). Asymmetric oviscapt twisting was observed in all mating couples in which the valves were visible, regardless of treatment (*n =* 47/66, Table 4, Additional file 3). To better visualize the asymmetrically twisted oviscapt valves in copulating females, we dissected three mating couples with untreated wild-type males by carefully removing the male parts. The position of the oviscapt valves was similar in these three mating couples. The valves were twisted clockwise and slightly spread apart. The medial gap between the valves, which was visible from the left side of the female, was narrow ventrally but rather broad dorsally and distally (Fig. 7b–d).

### Treated males and symmetric mutant males often fail to form a male–female genital locking system during copulation

**Fig. 8:**
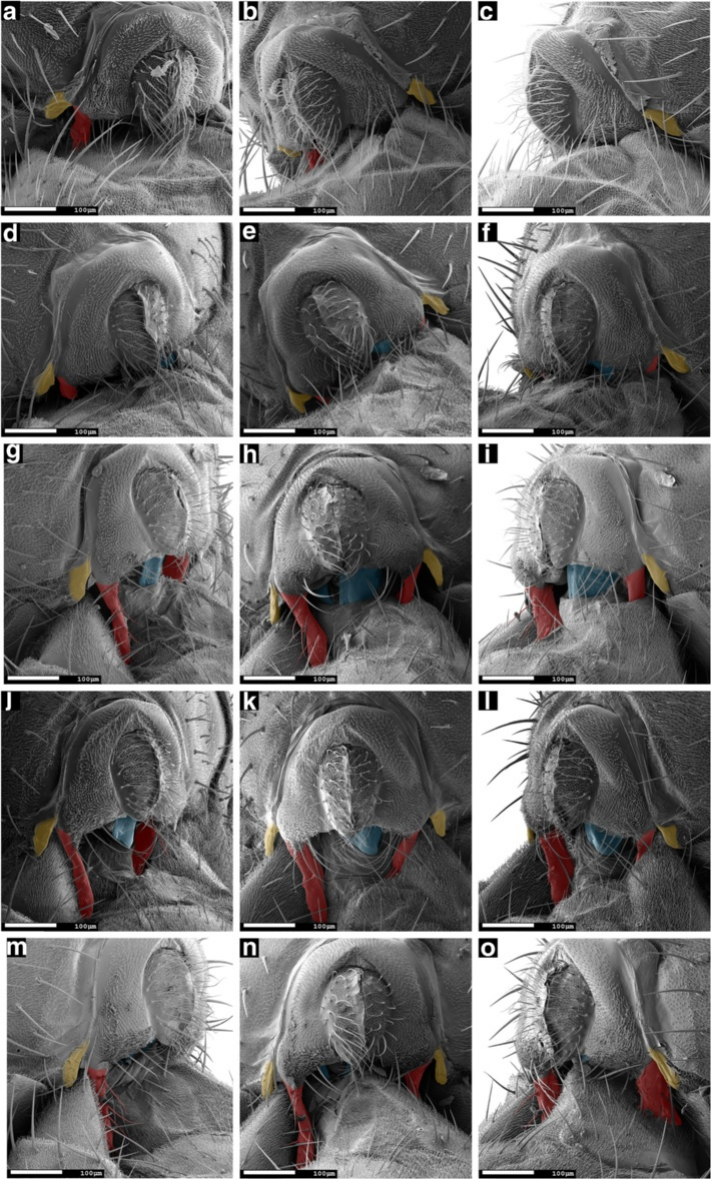
Males with surgically modified epandrial lobes and symmetric mutant males often cannot form a typical wild-type genital locking system. Scanning electron micrographs (190 × magnification) of male and female genitalia, 10 min into copulation, of a mating couple with a symmetric mutant male (**a**, **b**, **c**); a ‘left lobe cut’ male (**d**, **e**, **f**); a ‘right lobe cut’ male (**g**, **h**, **i**), a ‘left lobe shaved’ male (**j**, **k**, **l**) and a ‘right lobe shaved’ male (**m**, **n**, **o**). Views are left lateral (**a**, **d**, **g**, **j**, **m**), ventral (**b**, **e**, **h**, **k**, **n**) and right lateral (**c**, **f**, **i**, **l**, **o**). The male’s epandrial lobes are artificially coloured in *red*, the male’s lateral epandrial spines in *yellow* and the female’s oviscapt valves in *blue*. In (**a**–**f**), the left epandrial lobe is aberrantly placed dorso-laterally In (**g**–**i**, **m**–**o**), the right lobe is aberrantly placed dorso-laterally and in (**j**–**l**), both lobes are positioned as with unmodified wild-type males. Scale bar is 100 μm

To determine the effect of surgical removal of the left or right epandrial lobe on mating complex formation, we performed scanning electron microscopy of male–female mating complexes formed from ‘left lobe cut’ males (*n =* 14) or ‘right lobe cut’ males (*n =* 10). We observed that in 9 out of 14 cases, ‘left lobe cut’ males were not able to place the stump of their left epandrial lobe on the female’s ventral side near the ventral edge of the female’s 7th tergite, as unmodified wild-type males did (Table 4, Additional file 3). Instead, the remainder of the left lobe was placed more laterally or even dorsally on the female abdomen (Fig. 8d–f). In 3 out of 14 cases, ‘left lobe cut’ males did not place their (still intact) right epandrial lobe on the female abdomen as observed in couples with unmodified wild-type males. In such cases, the right epandrial lobe was not positioned on the female’s ventral side, and tightly pressed against the eversible sheath of the female’s oviscapt; instead, it was loosely positioned on the right side of the female abdomen, often on the dorsal side of the female’s 7th tergite. In one case, a ‘left lobe cut’ male positioned both epandrial lobes dorso-laterally on the female abdomen. Only 3 out of 14 ‘left lobe cut’ males placed their epandrial lobes as observed in mating couples with unmodified wild-type males. In summary, in most cases (11/14) ‘left lobe cut’ males could not form a genital locking system as observed in unmodified wild-type males. Therefore, the female’s 7th tergite could not be clamped between the male’s epandrial lobe and epandrial spine and the male’s epandrium was often positioned in a larger and more variable angle relative to the female abdomen (16.68 ± 10.45°, mean ± SD, *n =* 14; Table 4). The microscale angle between the female abdomen and the male’s epandrium of couples with ‘left lobe cut’ males was significantly larger than the microscale angle with unmodified wild-type males (Nemenyi’s post-hoc: *P =* 0.037).

‘Right lobe cut’ males were also found to fail forming a tight male–female locking system during copulation. In 5 out of 10 mating couples, the remaining stump of the right epandrial lobe was placed aberrantly dorso-laterally on the female abdomen, instead of being pressed against the female’s oviscapt (Fig. 8g-i, Table 4). One ‘right lobe cut’ male placed both of its epandrial lobes laterally (and not ventrally) on the female’s abdomen; this male’s epandrium was positioned in an extreme left-sided angle relative to the female. In the other 5 mating couples, both epandrial lobes were positioned as observed with untreated wild-type males: the left lobe was pressed on the female’s ventral side near the ventral edge of the female’s 7th tergite, and the stump of the right lobe closely pressed against the eversible sheath of the oviscapt. The microscale angle between the male’s epandrium and the female abdomen was estimated to be 1.15 ± 18.77° (mean ± SD, *n =* 10; Table 4) in mating couples with ‘right lobe cut’ males, which did not differ significantly from the angle between the male’s epandrium and the female abdomen in couples with unmodified wild-type males (Nemenyi’s post-hoc: *P =* 1.00). Overall, the angle between the male’s epandrium and the female abdomen was significantly affected by treatment (Kruskal-Wallis test: *K*_*5*_ = 14.62, *P =* 0.012).

Genital coupling in couples with symmetric mutant males resembled that of couples with ‘left lobe cut’ males. In 6 out of 16 mating couples with a symmetric mutant male, the right lobe was positioned ventrally on the female abdomen as in couples with unmodified wild-type males, but the left lobe was positioned aberrantly dorso-laterally on the female’s abdomen as observed in mating couples with ‘left lobe cut’ males. This resulted in the lack of a ‘lock’ between male and female genitalia (Fig. 8a-c). In 3 mating couples, the right lobe was positioned dorso-laterally instead of ventrally on the female’s abdomen, and the left lobe was positioned as observed in couples with unmodified wild-type males. In 1 out of 16 mating couples with a symmetric mutant male, both lobes were positioned aberrantly dorso-laterally on the female’s abdomen. The stereotyped male–female genital ‘lock’ as observed in couples with unmodified wild-type males occurred in 6 out of 16 mating couples with symmetric mutant males. The microscale angle between the male’s epandrium and the female’s abdomen was estimated to be 9.77 ± 12.36 (mean ± SD, *n =* 16; Table 4) in mating couples with symmetric mutant males, which was not significantly different from the angle between the male’s epandrium and female’s abdomen in couples with unmodified wild-type males (Nemenyi’s post-hoc: *P =* 0.670).

To investigate the role of epandrial lobe bristles in genital coupling, we examined mating couples with ‘left lobe shaved’ males (*n =* 4) or ‘right lobe shaved’ males (*n =* 8). Three out of four couples with ‘left lobe shaved’ males showed the stereotyped genital coupling that we found in couples with unmodified wild-type males (Table 4; Fig. 8j-l). However, in one ‘left lobe shaved’ mating couple the male placed its shaved (but uncut) left lobe dorsally on the female’s abdomen. Consequentially, this male was not able to grasp the female’s abdomen between its epandrial lobes and spines, and the male’s body was found in an extreme right-sided angle relative to the female.

In mating couples with ‘right lobe shaved’ males, the position of the lobes was the same as with unmodified wild-type males in 3 out of 8 cases (Table 4). In the remaining 5 cases, the right epandrial lobe was aberrantly dorso-laterally placed as observed in mating couples with ‘right lobe cut’ males (Table 4; Fig. 8m-o). In two of these ‘right lobe shaved’ mating couples with aberrantly placed right lobes, the tip of the left lobe was positioned on the female’s left side with the longitudinal axis of the lobe in an almost perpendicular angle to the female’s abdomen.

The effect of treatment on epandrial lobe placement during copulation was significant (omnibus Fisher’s exact test: *P <* 0.001, see Table 5 for *P*-values of post-hoc pairwise Bonferroni-corrected Fisher’s exact tests between treatments). To test whether epandrial lobe placement could explain the angle between male and female, regardless of treatment, we performed a post-hoc ANOVA with epandrial lobe placement as explanatory variable and the angle between male epandrium and female abdomen as response variable. Epandrial lobe placement, regardless of treatment, significantly affected the angle between the male’s epandrium and the female abdomen (*F*_*2,58*_ = 26.95, *P <* 0.001). Across treatments, couples in which the left lobe was aberrantly placed (*n =* 15) showed a more right-sided angle between male and female genitalia than couples in which both lobes were positioned in the stereotypical way observed in couples with unmodified wild-type males (*n =* 34; Tukey HSD: *P <* 0.001; Fig. 6b). This difference was also significant between couples with aberrantly placed left lobes and couples with aberrantly placed right lobes (*n =* 12; Tukey HSD: *P <* 0.001; Fig. 6b), but did not exist between couples with aberrantly placed right lobes and couples with stereotypically placed epandrial lobes (Tukey HSD: *P =* 0.525; Fig. 6b). Couples in which both epandrial lobes were aberrantly positioned on the female’s abdomen (*n =* 5) were excluded from this analysis, as these couples showed very variable and non-normally distributed angles between the male and the female.

**Table 5:** *P*-values of post-hoc pairwise comparisons of epandrial lobe placement in copulating *D. pachea* for treatments presented in Table 4

	Unmodified wildtype	Symmetric mutant	Left lobe cut	Right lobe cut
Unmodified wildtype	−			
Symmetric mutant	0.0041*	−		
Left lobe cut	0.0003*	>0.5	−	
Right lobe cut	0.0592	>0.5	>0.5	−
Right lobe shaved	0.0212*	>0.5	>0.5	>0.5

The category ‘left lobe shaved‘ was not included in the analysis because of the low number of observations obtained (*n =* 4). Fisher’s exact tests with Bonferroni corrections are used. *: *P <* 0.05

## Discussion

### *D. pachea* male anal plates are not involved in grasping the female

We found that male–female genital coupling in *D. pachea* is different from male–female genital coupling described in *D. melanogaster* and other species of the *melanogaster* species group. In the *melanog*aster species group, the male anal plates are actively involved in copulation and hold the female oviscapt distally together with the claspers which grasp the oviscapt valves [35, 38–40]. In these species, the dorso-ventral axes of male and female genitalia are inversely aligned, with male and female external genitalia touching over the complete posterior surface. This results in a male–female genital configuration in which the dorsal parts of the male genitalia make contact with the ventral side of the female genitalia and vice versa. In *D. pachea*, however, the anal plates do not touch the female during copulation and the dorso-ventral axis of the male epandrium is positioned perpendicular to the dorso-ventral axis of the female genitalia (Fig. 5a–d). Similar findings were shown by Eberhard and Ramirez for *Drosophila saltans*, *D. willistoni* and *D. malerkotliana* [60]*.* In these three distantly related species, which are each more closely related to the *melanogaster* species group than to *D. pachea* [61], the anal plates do not appear to participate in the genital complex either. Therefore, the involvement of the anal plates in copulation and the inverse configuration of male and female external genitalia described in *D. melanogaster* and other species of the *melanogaster* species group may be an exception rather than the norm among drosophilids. We found that in *D. pachea*, contact is mediated through the lobes, the phallus, the epandrial spines, and possibly the surstyli/claspers, which were not visible in the SEM micrographs because they were bent dorsally towards the female oviscapt valves and hidden by the male epandrium. However, we should note that our study only describes male–female genital coupling at 10 min after the start of copulation; it is thus possible that the anal plates participate in the genital complex in *D. pachea* at other time points during copulation. Scanning electron microscopy studies of genital complexes in other drosophilids are required to evaluate whether male anal plates are usually involved in copulation in *Drosophila*.

The configuration of the *D. pachea* genitalia complex can still be regarded as inversely correlated because the basis of the phallus is positioned with its dorsal side touching the ventral side of the female reproductive tract, and vice versa (Fig. 5). Nevertheless, the male and female genitalia are oriented relative to each other with an angle of about 6°, so that both genitals are not as perfectly aligned as in the other species of the *melanogas*ter subgroup [35, 38–40].

Interestingly, the differences in genital coupling between *D. pachea* and the *melanogaster* species group appear to be reflected in differences in the male-on-top mating position. During mating, the thoraxes of both male and female *D. pachea* are positioned parallel to each other, with the male on top and both heads pointing anteriorly (Fig. 3a). In the *melanogaster* species group, however, the male is positioned differently, with its head pointing upright and its thorax oriented perpendicularly to the female thorax, which is horizontally placed as in all *Drosophila* species during mating. Presumably, the inverse configuration of male and female genitalia in the *melanogaster* species group requires further bending of the male abdomen around the tip of the female abdomen than the configuration of male and female genitalia in *D. pachea*, in which the male anal plates are not involved. This could explain why the male mating position is upright in the *melanogaster* species group and horizontal in *D. pachea*.

### The right-sided mating position of *Drosophila pachea* is associated with asymmetric genital coupling

We observed that unmodified wildtype *D. pachea* males adopted asymmetric right-sided mating positions as described previously [43]: the male placed itself directly on top of the female between her wings, with his antero-posterior axis making a right-sided angle of 6.06 ± 3.64° (mean ± SD, *n =* 12) (macroscale angle) relative to the female antero-posterior axis. The present study provides, to our knowledge, the first detailed examination of how male and female genitalia contact each other in such fixed one-sided copulations in insects. We found that this asymmetric mating position was associated with a striking asymmetry in genital coupling: during copulation, the female’s oviscapt valves were twisted in a clockwise fashion and the male epandrium was positioned slightly asymmetrically relative to the female’s abdomen, with a stereotyped angle of 6.09 ± 4.40° (mean ± SD, *n =* 14). Importantly, this microscale angle is similar to the macroscale angle that we measured between male and female antero-posterior body axes in mating behaviour assays. The microscale angle observed in the genital complex is therefore maintained in the male posture on top of the female and is visible as an asymmetric placement of the male body with respect to the female midline. We thus suggest that the male abdomen does not twist laterally and that the macroscale mating angle reflects the microscale angle of the asymmetric genital coupling.

The female oviscapt valves were observed to be asymmetrically twisted during copulation with both unmodified (wildtype or mutant) males and treated males. Likewise, asymmetric right-sided mating positions were not only adopted by unmodified wildtype males, but also by surgically modified males in which the left epandrial lobe was ablated or shaved (present study) and by symmetric mutant males [43]. This shows that neither the twisting of the female’s oviscapt valves nor the adoption of an asymmetric mating position is directly caused by the presence of asymmetric epandrial lobes*.* The relationship between one-sided mating position and asymmetry in oviscapt twisting remains to be investigated. Possibly, the direction of mating towards the right side could be triggered by the female through female-induced oviscapt twisting. Alternatively, it could be determined by the male, but independently of the morphology of the lobe, for example through a left-right bias in the brain or in the eversion or morphology of the aedeagus. In that case, female oviscapt twisting could be facilitated or caused by the male-induced mating position.

### The lobes stabilize the asymmetric mating position

We found that the mating position was more variable in mating couples with males with a partially ablated or shaved left epandrial lobe than with unmodified wildtype males although it was always right sided. In particular, the large variance in mating angles in couples with ‘left lobe shaved’ males was largely due to a few extreme mating angles, whereas mating angles with ‘left lobe cut’ males were much more uniformly distributed. Similarly, mating positions were previously found to be more variable in symmetric mutant males than in wild-type males [43]. Furthermore, males with an ablated or shaved left epandrial lobe tended to adopt a mating position in which they were tilted to the female’s right side, whereas unmodified wildtype males and symmetric mutant males adopted a mating position right on top of the female. We observed that tilting males contacted the female’s wings, thorax or abdomen dorsally with their two anterior legs, presumably to ‘hold onto’ the female. Consequentially, tilted positions resulted in negative mating angles (Fig. 3b, Fig. 4).

In mating couples with unmodified wild-type *D. pachea*, the female’s 7th tergite was laterally ‘folded’ and firmly clamped between the male epandrial lobes and lateral epandrial spines. Consequentially, the female’s abdomen fit tightly between left and right epandrial lobes and left and right lateral epandrial spines. Symmetric mutant males and males with an ablated left or right epandrial lobe often positioned (the remaining stump of) their lobes aberrantly dorso-laterally on the female’s abdomen. As a result, the tight fit between the male’s epandrial lobes and spines and the female’s abdomen could often not form. Genital coupling with ‘right lobe shaved’ males was similar to genital coupling with ‘right lobe cut’ males (Table 4): in about half of the cases, the right epandrial lobe was positioned in an aberrant position laterally on the female’s body, resulting in a lack of genital locking. Aberrant lateral placement of the shaved right lobe sometimes caused the left lobe to be aberrantly positioned as well, which possibly affected the position of the male on the female in SEM assays. Together, these observations show that both left and right epandrial lobes are required for the establishment of a stable mating complex.

Aberrant placement of the left epandrial lobe resulted in a larger microscale angle between male and female genitalia compared to couples with stereotypically placed lobes, regardless of which treatment had caused the left lobe to be aberrantly placed. Aberrant placement of the right lobe did not consistently increase the angle between male and female genitalia, but aberrant placement of both epandrial lobes dramatically increased the variation in angles between male and female genitalia. Across treatments, in couples in which both epandrial lobes were stereotypically placed, the angle between male and female genitalia was similar to (and similarly variable as) this angle in couples with unmodified wildtype males. These observations show that both the left lobe and the right lobe need to be well positioned for the establishment of a stable mating complex, and that the lobe bristles play a sensory or physical role in positioning the lobes in the right way to form a stable mating complex. Therefore, we conclude that ‘left lobe cut’ and ‘left lobe shaved’ males, as well as symmetric mutant males [43], adopt atypical and sometimes unstable mating positions in behavioural assays because of a misplacement of the lobes and the lack of a tight fit between male and female genitalia. Importantly, not all ‘left lobe cut’, ‘left lobe shaved’ and symmetric mutant males in the behavioural assays adopted mating positions that were different from the wild-type.

In this study, copulation was achieved by almost all mating couples with ‘left lobe cut’ males, in contrast to our previous study [43] where 39 % of the symmetric mutant males were not able to copulate. In our video recordings, 17 % (3/18) of left lobe cut males and 11 % (2/18) of left lobe shaved males failed to mount the female at the first attempt while unmodified wildtype males never failed (0/16) (Additional file 1). In our previous study, the lobes of non-copulating mutant males were significantly smaller than those of copulating mutant males [43] and it was suggested that a critical lobe size might be required for efficient genitalia coupling. Indeed, the copulating ‘left-lobe cut’ males in this study had on average longer lobes than the non-copulating mutant males in our previous study (left: *T*_*22.03*_ = −2.83, *P =* 0.010; right: *T*_*9.14*_ = −7.43, *P <* 0.001), which corroborates this idea.

### Evolution of asymmetric lobes in *Drosophila pachea*

Epandrial lobes are not found in species that are closely related to *D. pachea* [45, 46], indicating that these asymmetric lobes evolved uniquely in the *D. pachea* lineage. Here we have tried to shed light on the function of these derived structures during copulation. We found that the epandrial lobes of *D. pachea* males are not essential for asymmetric genitalia coupling as males with amputated or shaved lobes still form asymmetric complexes and mate on the female’s right side, and so do mutant males with short symmetric lobes [43]. In addition, neither the twisting of the female’s oviscapt valves nor the adoption of an asymmetric mating position are directly caused by the asymmetry in epandrial lobes. Rather, our data show that the lobes stabilize the right-sided position of the male on top of the female and maintain a tight ‘lock’ of the female oviscapt during mating. The lobes might thus have evolved as an adaptation to stabilize the asymmetric configuration of male and female genitalia during copulation, thereby stabilizing the right-sided mating position.

Our experimental modification of epandrial lobes did not affect mating duration nor male resistance to female kicking behavior. This indicates that the lobes are not essential to maintain the mating complex or to drive the female into copulation. However, a critical minimal lobe size is required for efficient establishment of the mating complex in symmetric mutant males [43]. We also observed that lobe-modified males occasionally fail to mount the female. Under competitive conditions (which we have not tested) with multiple males courting and attempting to mate with a single female, asymmetric lobes might therefore be advantageous for quick and unfailing establishment of mate holding.

Asymmetric lobes may also enable the male to maintain a position in which sperm can be transferred efficiently into the female tract. If that is the case, the asymmetric mating angle could represent a particular emission angle at which sperm has a better chance, compared to a symmetric mating angle, to enter the uterus for post-copulatory transport into the female sperm storage organs, the spermathecae [62], to later fertilize eggs. In this scenario, males lacking asymmetric lobes would incur fitness costs because they cannot transfer sperm as efficiently as wild-type males into the female spermathecae. In this study we did not test whether the stereotyped wild-type one-sided mating in *D. pachea* generates more progeny than the aberrant mating positions that we observed. Future studies of sperm transfer in *D. pachea*, in normal conditions or with experimentally modified lobes, should shed further light on this important issue.

To our knowledge, no left-right morphological asymmetry is known in *D. pachea* females when they are in a non-copulatory state. However, the internal organs of *D. pachea* females have not been thoroughly investigated and it remains possible that females are also asymmetric. In bed bugs (Hemiptera: Cimicidae), asymmetric male intromittent organs [63] are associated with asymmetric internal female paragenitalia organs, also called ‘spermalege’ [64]. These female-specific organs appear to have an immunity and wound healing function [65] and to make traumatic insemination occur within a restricted area of the female’s abdomen. Their location varies between species: they can be either paired or one-sided, on the left or right side of the female abdomen [66].

Our data are consistent with the hypothesis proposed by Huber that an evolutionary change from an ancestral symmetric mating position to a fixed one-sided mating position would have caused an asymmetric contact zone between male and female genitalia, which would have favoured the evolution of genital asymmetry [1, 18]. Three non-mutually exclusive scenarios can be distinguished: (1) right and left sides of the genitalia may change to compensate for the mismatch resulting from asymmetric contact, so that the contact points between male and female is maintained on both sides; (2) right and left sides of the genitalia may start to assume different functions; (3) one side may lose any function and becomes reduced. Based on our observations, we can rule out scenario (3) because both lobes are required for the formation of a stable mating complex. We can also exclude scenario (1) because the left and right lobes contact different regions of the female oviscapt. Our study thus supports scenario (2): we found that the lobes contact different parts of the female genitalia bilaterally, and can therefore be considered as assuming different functions. Our detailed analysis of the function of *D. pachea* asymmetric lobes represents, to our knowledge, the first experimental investigation of genital complexes to test the hypothesis that the evolution of genital asymmetry is associated with changes in mating position [1, 18]. Our results are consistent with a novel genital asymmetry evolving in response to the evolution of one-sided mating, through the functional specialization of originally symmetric left and right genital organs. Future investigations of mating position and genitalia function in additional species, in the nannoptera group and in others, are now required to test Huber’s hypothesis further and to assess the generality of our results.

## Conclusion

Our study provides, to our knowledge, the first detailed functional analysis of male–female genital coupling in an insect species that displays a one-sided mating position. We found that the right-sided mating position of *D. pachea* is associated with asymmetric genital coupling. During mating, the female oviscapt is twisted asymmetrically and is grasped by the male’s asymmetric epandrial lobes in a tightly locked, stereotyped way. Consequently, the male epandrium is positioned at a slight angle relative to the female abdomen and the male adopts a right-sided mating position. Our analysis of mating positions of *D. pachea* males with surgically modified and unmodified epandrial lobes shows that the asymmetric lobes play an important role in stabilizing the spatial arrangement of male and female genitalia, thereby maintaining the position of the male on top of the female. The male anal plates, which grasp and hold the female during copulation in other *Drosophila* species, do not seem to be involved in copulation in *D. pachea*. Based on our results we conclude that *D. pachea* asymmetric epandrial lobes act as a stabilizing grasping device with distinct specialized functions on the left and right side.

## Methods

### Fly stocks

*Drosophila pachea* flies were retrieved from the San Diego Drosophila stock center (15090–1698.02) and maintained for about 40 generations at 25 °C in vials with 10 ml of standard Drosophila medium, mixed with 200 μg of 7-dehydrocholesterol (Sigma) dissolved in 40 μl of ethanol [67, 68]. In this stock, about 20 % of the males possess short and bilaterally symmetric epandrial lobes (symmetric males) [43]. All flies came from the 15090.1698.02 stock except a few symmetric mutant males which came from a selection line that was derived from the 15090.1698.02 stock by selecting symmetric males for several generations in order to increase the proportion of symmetric males.

### Epandrial lobe treatments

Newly emerged virgin flies were CO_2_-anaesthetized under a Stemi 2000 (Zeiss) stereo microscope using a CO_2_-pad (INJECT + MATIC sleeper) and were isolated. Males were transferred into individual vials and virgin females of the same age into groups of 5–20 individuals per vial. After sorting, males were left to mature for at least 14 days and females for at least 4 days. The epandrial lobe treatment of wild-type males took place at room temperature at 7 to 9 days after they eclosed from the pupa, when the males were not yet sexually mature [43, 67, 69].

For the surgical lobe treatments with micro-scissors (‘left lobe cut’ and ‘right lobe cut’), each male was CO_2_-anaesthetized as described above and immobilized on its back by pressing a small looped copper wire on the male’s abdomen. Then part of the male’s left or most of the right epandrial lobe was cut off using 9600-Vannas scissors (Moria). All males survived this treatment. The ‘unmodified’ treatment was the same, except that no epandrial lobe was cut. Males were left to recover isolated in single vials for at least 3 days prior to the mating experiments.

For laser ablation treatments (‘left lobe shaved’ and ‘right lobe shaved’), each male fly was CO_2_-anaesthetized and placed into a custom-made laser-ablation chamber (Additional file 4), adapted from the one described by Rashed & Polak 2010 [51]. The ablation chamber was mounted on a Ti-E inverted microscope (Nikon) that was associated to a spinning disc confocal head (CSU-X1; Yokogawa Corporation of America) or to a IX83 Inverted Microscope (Olympus), both equipped with a 491-nm laser (Roper Scientific). Flies were observed and bristles were laser-ablated at 400x magnification. Acquisition and laser parameters were controlled by MetaMorph software (Molecular Devices) and iLas^2^ software (Roper Scientific). Laser parameters were set to 15–23 % laser power and 500–1000 spots. Removal of all the bristles of an epandrial lobe required about 30 laser pulses (one pulse per bristle) and took 5–10 min. All males survived the laser surgery treatment. After laser surgery, males were isolated in vials and left to recover for at least 4 days prior to the mating experiments.

### Video recordings of mating behaviour

Mating behaviour was assessed in mating couples with unmodified males, ‘left lobe cut’ males and ‘left lobe shaved’ males, and was recorded following the protocol described in [43]. A virgin male fly and a virgin female fly were introduced into a small circular plastic cell (diameter 10 mm, height 4 mm) that was covered by transparent 1 mm Plexiglas. Virgin females were between 4 and 26 days old, with a median age of 9 days. The couple was filmed from above with a digital camera (either a 5-Megapixel USB DigiMicro Profi (DNT) camera or a Conrad 2-Megapixel USB/Flat 2 Mio digital microscope camera, Nr 191251) until copulation ended. Video recordings were done in a temperature-and humidity-controlled chamber at 25 °C (±0.5 °C) and 60 % (±2 %) humidity. We gathered 52 recordings in total: 18 with ‘left lobe shaved’ males, 18 with ‘left lobe cut’ males and 16 with unmodified males.

### Measures of mating behaviour

To measure the mating position angle, screenshots of the video recordings were extracted approximately every 2.5 min from the start of copulation until the end of copulation, but only when the female was viewed exactly on its dorsal side. On each screenshot, a line was drawn between the bases of the wings in both the male and the female, and the angle between the two lines was measured using tpsDig 2.17 software [70]. The resulting angle is equivalent to the angle between the male’s and the female’s antero-posterior body axis as measured in our previous study [43] (Fig. 3a). This new method makes it possible to estimate the angle even when the male is tilted towards one side of the female whereas the previous method does not.

**Fig. 9:**
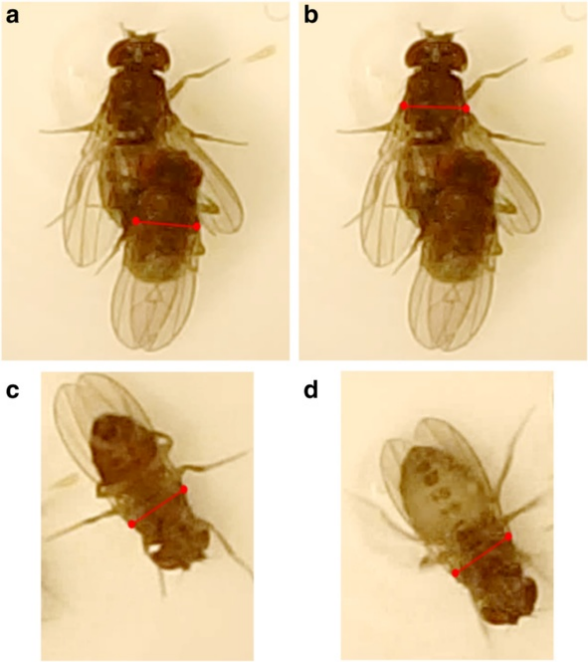
Measurement of tilting index parameters. Tilting index TI_i_ (*t*) at a certain time point *t* in mating couple *i* was calculated by measuring the following parameters: **a** distance between the bases of the wings in the male during copulation *d(M*_*i*_ *,t)*, **b** distance between the bases of the wings in the female during copulation *d(F*_*i*_ *,t)*, **c** distance between the bases of the wings in the male in resting position *d(M*_*i*_ *, restingposition)* and **d** distance between the bases of the wings in the female in resting position *d(F*_*i*_ *, restingposition)*. Note that measurements of flies in resting position were taken when they were positioned upside-down on the Plexiglas cover of the mating cell, to be certain that the flies were positioned on a horizontal surface

On each screenshot, the degree to which the male was tilted towards the female’s right side was assessed through an index defined as the tilting index. If *d (M*_*i*_*,t)* is the distance measured (in number of pixels) between the bases of the left and right wing of a male *M* in mating couple *i* at time *t* into copulation, and *d (F*_*i*_*,t)* is the distance measured between the bases of the left and right wing of a female F in mating couple *i* at time *t* into copulation, then the tilting index *TI* at time *t* is defined as

$$ T{I}_i(t)=\frac{d\left({M}_i,t\right)/d\left({F}_i,t\right)}{d\left({M}_i, restingposition\right)/d\left({F}_i, restingposition\right)} $$

where ‘resting position’ means a position before or after copulation in which the male or the female was positioned upside down on the Plexiglas cover of the mating cell, and therefore viewed exactly ventrally (Fig. 9). In this resting position, the bases of the wings were always clearly visible and the distance between the wings was maximal.

The tilting index ranges between 0 and 1, with values approximating 1 when the male is not tilted relative to the female and values decreasing as the male is ‘sliding off’ downwards on one side of the female. Values that slightly exceed 1 occurred occasionally and were due to the female not being observed in perfect dorsal view during mating.

We measured several additional parameters: the time before male courtship initiation (defined as the start of wing-flicking behaviour), the duration of courtship until the beginning of copulation (defined as the first moment when the male starts mounting the female in a successful manner, that is, after this mounting, the male will stay on top of the female for more than 1 min), the number of unsuccessful mounting attempts of a male (defined as events where the male stayed on top of the female for less than a minute), the total duration of mating (from the beginning of copulation to the moment the male is detaching itself from the female), the time from the beginning of copulation until the female starts kicking the male’s abdomen with her hind legs, and the remaining duration of mating after the female had started kicking.

### Statistical analyses of mating behaviour

All statistical analyses were done using R v. 3.1.0 [71]. We investigated the effect of treatment (left lobe cut, left lobe shaved, and unmodified male) on mating parameters using linear models with identity link functions or Kruskal-Wallis tests if the assumptions of homoscedasticity and normality were violated. Variables were log-transformed if that improved model diagnostics.

We assessed differences in mating angle and tilting index between treatments after 10–15 min of copulation, when the variation in mating angles with unmodified males was lowest. If there were multiple observations within that time frame for a particular mating couple, we took the mean of those observations. Tilting indices were bounded between 0 and 1 and were arcsine-transformed before statistical analysis. The arcsine transformation was used to transform tilting index, which is a projection of the angle of tilting in the horizontal plane, into a value that corresponds to the actual tilting angle. Values that slightly exceeded 1 due to measurement error were taken to be 1 for statistical analysis.

For mating angle and tilting index, we assessed differences in variances between treatments using Bartlett’s test of variance homogeneity. If Bartlett’s test was significant, we used post-hoc F-tests with strict Bonferroni correction for pairwise comparisons of variances between treatments. We assessed differences in means between treatments using generalized least squares linear modelling; we included a variance covariate per treatment if the variances were not homogeneous. We used Tukey’s post-hoc test (if no variance covariate was included in the model), or pairwise t-tests with Bonferroni corrections and non-pooled standard deviations (if a variance covariate was included in the model) to do pairwise comparisons of means.

We tested whether mating angle after 10–15 min of copulation was correlated with tilting index after 10–15 min of copulation, using correlation tests for each treatment (left lobe cut, left lobe shaved, and unmodified male) separately. We also tested whether mating angle and tilting index were correlated with tibia length (as a proxy for body size), left epandrial lobe length, right epandrial lobe length, the sum of left and right lobe lengths or the ratio of lobe lengths. We used Spearman’s rank correlation tests rather than Pearson’s product–moment correlation tests, as the latter are sensitive to outliers in small datasets.

### Genitalia dissection for light microscopy

After video recording, the males were stored in absolute ethanol at − 20 °C and then rehydrated for 5 min in phosphate buffer saline (PBS). Subsequently, the epandrium and the left foreleg were dissected using fine needles under a Zeiss Stemi 2000 stereo microscope. The dissected epandria and the legs were then washed twice in 100 % glycerol and mounted in 100 % glycerol. Dissections were imaged at 200x magnification using a Keyence VHX2000 microscope with a 100–1000× VH-Z100UR/W lens. Measurements of left and right epandrial lobe lengths (Fig. 1a) and left foreleg tibia length were done using tpsDig 2.17 software [70].

### Scanning electron microscopy (SEM) of mating couples

Single virgin male flies (unmodified wild-type, ‘left lobe cut’, ‘right lobe cut’, ‘left lobe shaved’, ‘right lobe shaved’ or symmetric mutant) were introduced with single virgin female flies in Eppendorf tubes. These tubes were roughened on the inside with a Dremel 3000 and a 5 mm grinding head, as smooth surfaces of unprocessed Eppendorf tubes prevented the flies from attaching to the walls of the tubes, thereby preventing mating. In most cases, mating occurred within 90 min after the introduction of the male. At exactly 10 min after the beginning of copulation, couples were flash-frozen by submerging the Eppendorf tubes into liquid nitrogen for at least 20 s. Subsequently, tubes were filled up with cold (−20 °C) absolute ethanol and quickly transferred to a − 20 °C freezer, where samples remained for at least a week. The frozen and fixed couples were placed in 80 % ethanol at room temperature. This method makes it possible to study the positions of male and female genital structures during copulation, at the exact moment when the couple was frozen [60]. In total, we collected 77 mating couples in six treatments.

**Fig. 10:**
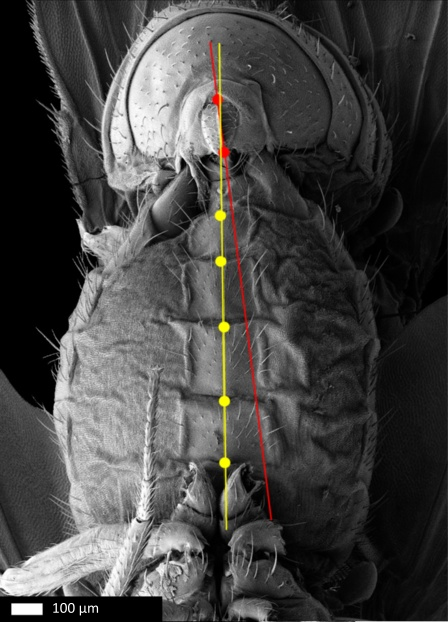
Measurement of the microscale angle between male epandrium and female abdomen during copulation. SEM of a *D. pachea* mating complex (37-fold magnification) in ventral view (Fig. 5a). The angle between the male and the female was measured as the angle between a line along the dorso-ventral axis of symmetry of the male’s epandrium (*red*) and a second line connecting the mid-points of the female’s sternites (*yellow*). Scale bar is 100 μm

Mating couples were critical-point dried using an EM CPD300 automated critical point dryer (Leica), then mounted on aluminium stubs with the female’s ventral side facing upwards and coated with platinum/palladium (20 nm). Each mating couple was SEM-imaged with a JSM-7500 F field emission scanning electron microscope (Jeol) in female ventral view at 37×, 75×, 110×, 150×, 190×, 250× and 350× magnification, and in lateral view (left and right) at 75× and at 190× magnification. For each mating couple, the positions of the epandrial lobes and lateral spines on the female was observed. We measured the angle between the male’s and the female’s antero-posterior body axis using tpsDig 2.17 software [70], by assessing the angle between the epandrial dorso-ventral axis and a straight line going through the midline points of the female’s sternites (Fig. 10).

During copulation, female external genital structures are partially covered by the male genitalia. To dislodge the mating male and thus completely visualize female structures, three critical-point dried and previously SEM-imaged mating couples were dissected. The uncovered female genital structures were then re-examined under the SEM procedure described above.

### Statistical analyses of genital coupling

All statistical analyses were done using R v. 3.1.0 [71]. For each treatment, we classified mating couples based on their SEM images into two categories: mating couples in which the male genitalia were coupled with the female genitalia in a stereotyped ‘unmodified wild-type’ way, and mating couples in which one or both epandrial lobes were aberrantly placed. In the resulting contingency table we looked for an overall effect of treatment on genital coupling using a two-sided Fisher’s exact test. We subsequently used post-hoc Fisher’s exact tests with Bonferroni correction to assess the significance of pairwise differences in lobe placement between treatments.

To test if aberrant epandrial lobe usage results in aberrant mating positions, we performed a post-hoc ANOVA with epandrial lobe placement as explanatory variable (with three states: normal, left lobe aberrantly placed and right lobe aberrantly placed) and the angle between male epandrium and female abdomen as response variable. We used Tukey’s post-hoc test to do pairwise comparisons of means.

We looked for an effect of treatment on the angle between the male’s epandrium and the female’s antero-posterior body axis using the non-parametric Kruskal-Wallis test, as the requirements of homoscedasticity and normality for parametric testing were not met. We used Nemenyi’s post-hoc test from the R package *PMCMR* [72] for post-hoc pairwise comparisons of medians to assess the significance of differences between treatments.

## Additional files

Additional file 1: Raw measurements of mating behaviour, mating positions and genitalia. (XLSX 62 kb) Additional file 2: Measurements of mating behaviour. (XLSX 18 kb) Additional file 3: Measurements of SEM analysis. (XLSX 11 kb) Additional file 4: Supplementary method. Description of the laser ablation chamber. (PDF 681 kb)

## Abbreviations

LM: Linear model
SD: Standard deviation
SEM: Scanning electron microscopy
