# Supplementary material for: Drosophila pachea asymmetric lobes are part of a grasping device and stabilize one-sided mating
Source: BMC Evol Biol. 2016 Sep 1;16(1):176. doi: 10.1186/s12862-016-0747-4 (PMC5009675; doi:10.1186/s12862-016-0747-4)
Supplement: Additional file 4: — Supplementary method. Description of the laser ablation chamber. (PDF 681 kb) [file 12862_2016_747_MOESM4_ESM.pdf]

Supplementary Methods

Laser ablation chamber description

This chamber consisted of a modified POC-R2 Cell Cultivation System (PECON) for 32 mm Ø round cover slips (0.1 mm thick), in which the chamber was replaced by a plastic ring (upper 15 mm of a Maxiprep column (PureYield™ Plasmid Maxiprep System (Promega)). A tube connector was fitted into this ring, and a 5 mm Ø silicon tube was connected for CO_2 supply. A tap in the tube was used to regulate the CO_2 flow into the chamber. The fly was immobilized while standing on its legs on the cover slip of the ablation chamber by carefully adjusting a plastic grid on top of its body: a 500 micron plastic mesh (NYTAL 1-002-M-SEFAR) glued to the 'upper' part (15 mm) of a 20-mL syringe cylinder (Fig. S1B-C). For the grid to fit better into the ablation chamber (items 7 and 2 in Fig. S1), an extra layer of plastic (item 7b in Fig. S1B) taken from the "upper" external part (5 mm) of a 20-mL syringe was glued around the mesh-cylinder (item 7 in B) to increase its diameter. The width of the grid was also fine-adjusted with tape to fit (with slight resistance) into the ablation chamber. A notch was present in the grid to allow for the tube connector to fit into the mating chamber. This tube connector had the opening facing downwards in a 90˚ angle, to allow CO_2 to stream to the bottom of the ablation chamber. The ablation chamber was connected via a CO_2 regulator (INJECT+MATIC sleeper) to a pressure controller (0.5-1 bar) of a CO_2 bottle.

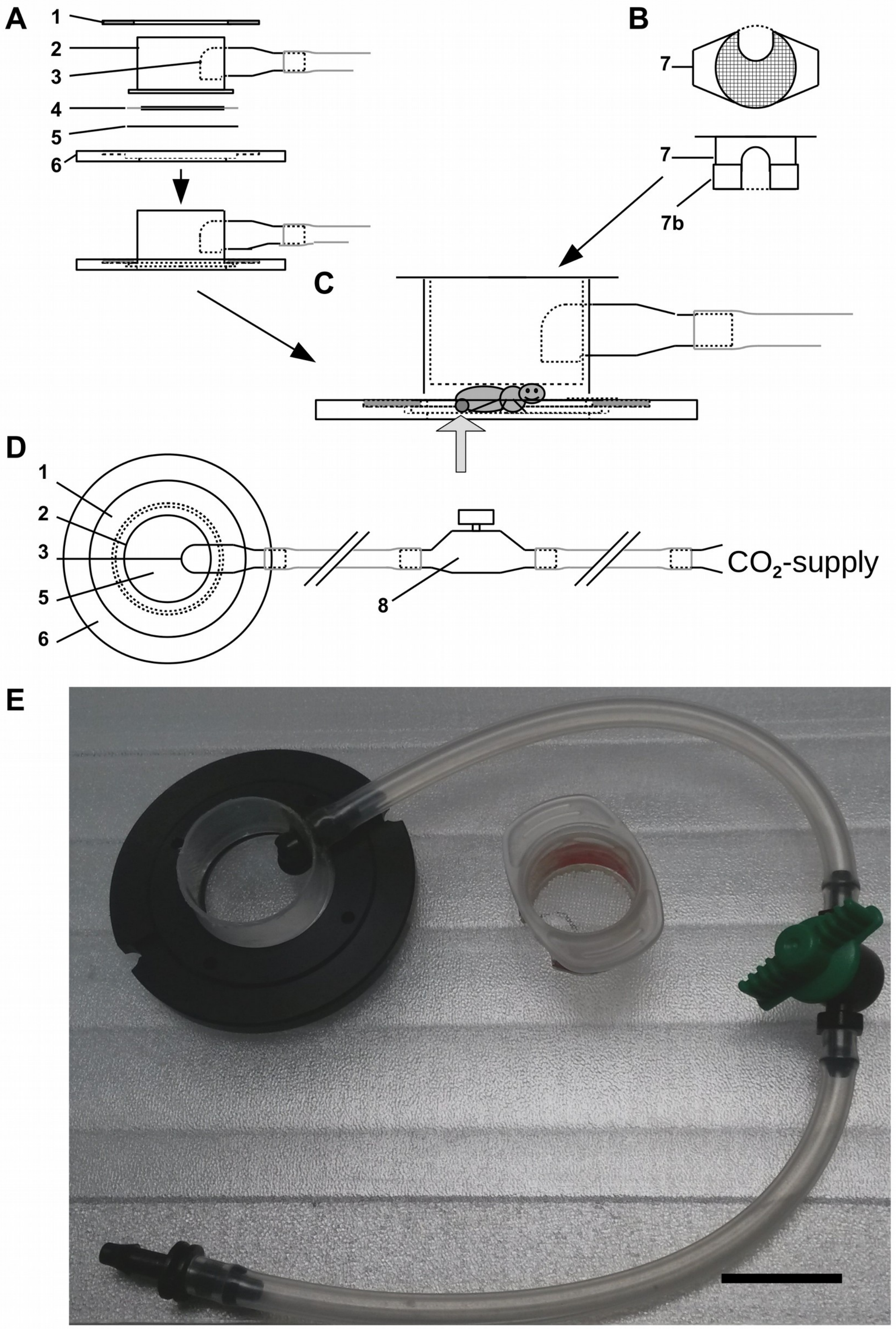

Figure S1: Laser-ablation set-up. *(A) Ablation chamber viewed from the side, with single parts disassembled and assembled (below the arrow) 1: POC-R2 Cell Cultivation System (PECON) 45 mm screw-ring to press the cover slip and the ablation device onto the ground support; 2: Plastic ring (upper 15 mm of a Maxiprep column (PureYield™ Plasmid Maxiprep System (Promega)); 3: Tube connector (gardening supply) for 5 mm silicone tubes with the opening facing downwards; 4: Silicone ring of the POC-R2 Cell Cultivation System (PECON); 5: Round Ø 32 mm cover slip; 6: POC-R2 Cell Cultivation System (PECON) 60 mm ground support. (B) Removable grid to immobilize flies on the cover slip, 7: plastic mesh, 500 micron (NYTAL 1-002-M-SEFAR) glued to the "upper" external part (10 mm) of a 20-mL syringe (Ø ~ 24 mm) cylinder, 7b: outer plastic ring. (C) Overdimensioned fly in the ablation chamber as an illustration of the set-up. The direction of the laser beam is indicated by the grey arrow. (D) Ablation chamber viewed from above, 8: tap (gardening supply) to regulate the CO_2 flow. (E) Photo of the ablation chamber. Scale bar is 25 mm.*
